# Binding Sites of Anti-Lcr V Monoclonal Antibodies Are More Critical than the Avidities and Affinities for Passive Protection against *Yersinia pestis* Infection in a Bubonic Plague Model

**DOI:** 10.3390/antib9030037

**Published:** 2020-08-03

**Authors:** Kei Amemiya, Jennifer L. Dankmeyer, Sarah L. Keasey, Sylvia R. Trevino, Michael M. Wormald, Stephanie A. Halasohoris, Wilson J. Ribot, David P. Fetterer, Christopher K. Cote, Patricia L. Worsham, Jeffrey J. Adamovicz, Robert G. Ulrich

**Affiliations:** 1 Bacteriology Division, United States Army Medical Research Institute of Infectious Diseases, Fort Detrick, MD 21702, USA; Jennifer.l.dankmeyer.ctr@mail.mil (J.L.D.); sylvia_trevino@comcast.net (S.R.T.); mwormal1@jhu.edu (M.M.W.); stephanie.a.halasohoris.ctr@mail.mil (S.A.H.); wilson.j.ribot.civ@mail.mil (W.J.R.); david.p.fetterer.ctr@mail.mil (D.P.F.); christopher.k.cote.civ@mail.mil (C.K.C.); worsham@fred.net (P.L.W.); adamoviczj@missouri.edu (J.J.A.); 2 Molecular and Translational Sciences, United States Army Medical Research Institute of Infectious Diseases, Fort Detrick, MD 21702, USA; keasey1@umbc.edu (S.L.K.); rulrich@bhsai.org (R.G.U.)

**Keywords:** monoclonal antibodies, *Yersinia pestis*, plague, binding sites, avidities, affinities, passive protection

## Abstract

Plague is a zoonotic disease that is caused by *Yersinia pestis*. Monoclonal antibodies (mAbs) that bind to the V-antigen, a virulence factor that is produced by *Y. pestis*, can passively protect mice from plague. An analysis of protective mAbs that bind to V-antigen was made to assess binding sites, avidities, and affinities. Anti-V mAbs were screened for their efficacy in a murine model of plague. Antigen-binding sites of protective V mAbs were determined with a linear peptide library, V-antigen fragment, competitive binding, and surface plasmon resonance. The avidities to the V-antigen was determined by ELISA, and affinities of the mAbs to the V-antigen were determined by surface plasmon resonance. The most protective mAb 7.3 bound to a unique conformational site on the V-antigen, while a less protective mAb bound to a different conformational site located on the same V-antigen fragment as mAb 7.3. The avidity of mAb 7.3 for the V-antigen was neither the strongest overall nor did it have the highest affinity for the V-antigen. The binding site of the most protective mAb was critical in its ability to protect against a lethal plague challenge.

## 1. Introduction

One of the most devastating zoonotic diseases in history was caused by the bacterium *Yersinia pestis* in the 14th century, which may have caused the death of 25–30 million people in Europe [1,2]. Normally a disease of the rat and other rodents, the bacterium can be transmitted to humans by the bite of an infected rat flea or other human ectoparasites causing bubonic plague. It has been suggested [2] that *Y. pestis* was derived from *Yersinia pseudotuberculosis*, a closely related human pathogen that causes gastroenteritis, partially by the loss of approximately 1.5% of its genetic material and the acquisition of a 100 kb plasmid (pMT1) that carries the *caf*1 (F1 protein) and murine toxin, and a 10 kb plasmid (pPCP1) that encodes the plasminogen activator gene and other genes. These genetic alterations allow *Y. pestis* to infect mammalian hosts by subverting the host immune response to the pathogen. All pathogenic *Yersinia* sp., however, still share a common 70 kb plasmid (pCD1) that encodes proteins of a type III secretion system (T3SS) that is necessary to alter the immune response of the host cell during pathogenesis. [2,3]. The T3SS is one of a number of types of injectisomes that are used by pathogenic Gram-negative bacilli to mediate infection of animal and plant cells [4]. The tip structure of the needle-like injectisome of the T3SS is formed by the *Yersinia* outer membrane proteins (Yops) YopB and YopD, and the low calcium response V-antigen (LcrV) [5]. Contact with the host cell membrane drives the injection of additional Yop effector proteins into the cell through the injectisome to increase host susceptibility to infection by a variety of mechanisms.

Alexandre Yersin and Shibasaburo Kitasato first observed the plague bacilli in Hong Kong in 1894, although Yersin was given credit for the discovery [6]. Two years later, Yersin was able to show efficacy of immune antisera against plague in humans. In 1897 Haffkine used a heat-killed preparation of *Y. pestis* to protect rabbits, demonstrating the first plague vaccine [6]. Heat-killed bacilli were used for vaccination until 1931 when an attenuated live plague vaccine was developed. However, the attenuated live vaccine was highly immunoreactive and is currently used only in Eastern Europe. A formalin-inactivated plague vaccine was developed by Meyer’s group in the late 1930s, and used by the U.S. Army until the 1990s, but it was discontinued because it did not protect against an aerosol exposure to plague [6]. A primary protective antigen in the inactivated whole cell plague vaccine was the F1 capsule protein. It was not until the 1950–1960s that Meyer’s group [7] and Lawton et al. (1960, 1963) [8,9] showed that biochemically purified F1 protein and V-antigen, which were considered to be virulence factors, could offer protection against plague in vaccinated mice and nonhuman primates. Later it was shown by Williamson et al. (1995) [10] that a combination of a biochemically purified F1 protein and recombinant V-antigen provided more protection than the individual proteins in a plague animal model. Subsequently, a recombinant fusion protein that consisted of the F1 and V-antigen (F1-V) was shown to be protective in both bubonic and pneumonic plague animal models [11,12]. Although there is no human vaccine against plague that is approved by the Food and Drug Administration for general use, vaccines consisting of F1 + V or F1-V have been in early Phase 1 and II clinical trials, respectively.

There was a significant correlation with levels of IgG1 or IgG induced by F1 + V or F1-V, respectively, and protection in a mouse model of plague [13,14]. For the case of F1-V, CpG oligodeoxynucleotides were used to enhance the host humoral response to the vaccine in a murine model of plague, and this study demonstrated that there was a significant increase in probability of survival with an increase in IgG to the vaccine [14], suggesting that immune protection was associated with antibody responses. Cell-mediated immunity [15,16] and the innate immune responses [17] may also play a role in protection against pneumonic plague in F1 – V vaccinated mice. A central role for antibodies in the protection against plague is further supported by studies with monoclonal antibodies (mAbs) against F1 or V antigen that mediated protection against a lethal challenge [18,19,20]. Hill et al. (1997) [19] had initially reported that the protective V antigen mAb 7.3 recognized a conformational site encompassing amino acids 135–275 of the V antigen and was further defined to include amino acid residue N255 [21]. Quenee et al. (2010) [20] reported that the protective mAb BA5 bound to a region of the V antigen that encompassed amino acids 196–225, which was also within the region (135–275 amino acids) of the binding site for mAb 7.3. When mAb 7.3 and BA5 were compared, 7.3 appeared to be more potent based on the amount of anti-plague mAb needed (35 µg vs. 200 µg, respectively) to protect a majority of mice exposed to the plague bacterium [20,22]. The questions addressed in the study described here are: was the potency of mAb 7.3 attributable to the avidity or affinity of the mAb to the V antigen or possibly the recognition site on the V-antigen? A number of anti-V antigen mAbs were isolated to evaluate their use in the therapeutic treatment against *Y. pestis* infection, and their use as an internal standard in a competitive ELISA. We examined how the interaction of these anti-V mAbs with the V-antigen compared with that of the protective mAb 7.3. Thus, the binding of the anti-V-antigen mAbs on the V-antigen was first examined with linear peptides that spanned the V-antigen and a fragment of the V-antigen to try and map the binding sites of the mAbs including that of 7.3. Further, the avidity and affinity of the anti-V mAbs for the V-antigen were evaluated with that of mAb 7.3. Our results suggest that it may be partly the specific binding site of mAb 7.3 on the V-antigen and the consequences (directly or indirectly) from its binding to that site and not the avidity or affinity of the mAb for the V-antigen that was responsible for its efficacy.

## 2. Materials and Methods

### 2.1. Bacterial Strains and mAbs

Bacterial strains, *Y. pestis* CO92 [23] and *Y. pestis* C12 [24] were obtained from the bacterial collection in the Bacteriology Division at the US Army Medical Research Institute of Infectious Diseases (USAMRIID), and they were grown as previously described [25,26]. The anti-V-antigen mAbs (cells and cell culture supernatants) with their isotypes were obtained from Battelle (Battelle, Columbus, OH, USA). The selected mAb producing hybridoma cells were further expanded by the Cell Culture Division at USAMRIID, and the mAbs were purified by Dr. Bill Gillette (NCI, Frederick, MD, USA) using a protein G column. The mAb 7.3 (IgG1) was a kind gift from Dr. Jim Hill (Defence Science and Technology Laboratories, Porton Down, Salisbury, Wiltshire SP4 OJQ, United Kingdom). Other positive control or negative control serum or mAbs were obtained from the Bacteriology Division at USAMRIID.

### 2.2. Animal Studies

Female, 6–8 weeks old BALB/c or Swiss Webster mice were obtained from the National Cancer Institute (Frederick, MD, USA). Mice were housed in clean, filtered top pans, with mouse litter, an enrichment item, and given water and food ad libitum, and mouse litter and pans were changed twice a week. For passive protection studies, mice were given the stated amount (see Tables or Figure Legends) intraperitoneal (i.p.) 24 h before challenge. The challenge dose was delivered either subcutaneous (s.c.) for a bubonic plague model (1 LD_50_ is ~2.0 CFU, [27]) or by whole-body aerosol (1 LD_50_ is 6.8 × 10^4^ CFU, [28] for a pneumonic plague model. After challenge mice were observed twice daily for the period stated in the appropriate tables or figures.

Research was conducted under an IACUC approved protocol in compliance with the Animal Welfare Act, PHS Policy, and other federal statutes and regulations relating to animals and experiments involving animals. The facility where this research was conducted is accredited by the Association for Assessment and Accreditation of Laboratory Animal Care, International and adheres to principles stated in the 8th Edition of the Guide for the Care and Use of Laboratory Animals, National Research Council, 2011.

### 2.3. Binding of mAbs to V-Antigen Peptides or V-Antigens

A standard ELISA assay was used with slight modifications to examine the binding of mAbs to peptides, V-antigen fragment or V-antigen in 96-well Immunlon 2 HB plates (Thermo Fisher Scientific, Waltham, MA, USA) [29]. The V-antigen 53 peptide array library was obtained from the Biodefense and Emerging Infections (BEI) Research Resources Repository (American Type Culture Collection, Manassas, VA, USA), dissolved in DMSO (Sigma/Aldrich, St. Louis, MO, USA) and used at 25 µg/mL for examining the binding of the anti-V-antigen mAbs. The V-antigen was purified as previously described and was used at 10 µg/mL [12]. The 135–275 amino acid fragment of the V-antigen was obtained from Dr. Bill Gillette (National Cancer Institute, Frederick, MD) and was used at 10 µg/mL. The antigens were diluted to the concentrations listed above in 0.1M carbonate buffer, pH 9.5, to the concentrations stated above and used to coat 96-well plates at 50 µL per well in triplicate, and the plates were incubated overnight at 4 °C. After washing the plates, they were blocked, and samples processed as previously described [29]. To measure the amount of binding of the mAbs to the antigens, anti-mouse IgG conjugated with horseradish peroxidase (HRP) was used (Southern Biotech, Birmingham, AL, USA) and color read at 450 nm.

### 2.4. Competitive Binding of mAbs

In order to examine the competitive binding of mAbs to the V-antigen we needed a biotinylated mAb preparation to distinguish bound from unbound mAb. Thus, an aliquot of each mAb was buffer-exchanged into 50 mM sodium carbonate buffer, pH 8.5, with an Amicon 30 kDa centrifugal filter unit (MilliporeSigma, Burlington, MA, USA). A portion of the mAb preparation was saved and used as the respective unlabeled mAb. One mg of NHS-LS-Biotin (ThermoFisher, Waltham, MA, USA) was dissolved in 50 mM sodium carbonate, pH8.5, to make a 1 mg/mL solution. Biotin was added at a ratio of 1:20 with the mAb, and the mixture was incubated on ice for 2 h. Excess biotin was removed by size exclusion filtration with an Amicon 30 kDa filter unit. The protein concentration of the biotinylated mAb was determined by the Bradford Assay method. An ELISA with V-antigen coated 96 well plates was used to determine the high (90%) and low (70%) binding concentration of the biotinylated antibody. When the mAbs are biotinylated, the secondary or detection antibody was a streptavidin-HRP (Southern Biotech, Birmingham, AL, USA) to measure the amount of biotinylated mAb bound to the V antigen.

For competitive binding ELISA assays (mAbs 141.1 vs. 7.3) the ELISA plates were coated with V-antigen, 2 µg/mL, in 0.1M carbonate buffer, pH 9.5 and incubated overnight at 4 °C. Plates were washed with 1X PBS-T Wash Buffer (1X Phosphate Buffered Saline, pH 7.4, 0.05% Tween 20) and blocked for 1 h with 1% Blocker Casein in 1X PBS (ThermoFisher, Waltham, MA, USA). After incubation, plates were washed and either unlabeled anti-V mAbs 141.1 or 7.3 or biotinylated anti-V mAbs 141.1 or 7.3 were added at 0.1 µg/mL and incubated for 2 h at 37 °C. The plates were then washed and treated with streptavidin-labeled HRP and incubated for 1 h at 37 °C. After a final wash, the plates were developed and read as previously reported [29].

### 2.5. Avidity ELISA Assay

The avidity (or overall strength in binding) of the anti-V mAbs interacting with the V-antigen was evaluated by ELISA in the presence of increasing concentrations of ammonium thiocyanate. ELISA plates were coated with 10 µg/mL of V-antigen in 0.1 M carbonate buffer, pH 9.5, and stored overnight at 4 °C. The plates were washed with 1X PBS-T and blocked with casein for 1 h. After washing, 100 pg per well of each anti-V mAb was added in triplicate wells, and the plates were incubated for 1 h at 37 °C. Separately, ammonium thiocyanate was serially diluted starting at 8 M to 0.015 M, and diluted ammonium thiocyanate was added to the V- antigen-mAbs complex in the triplicate wells. The plates were incubated for an additional 30 min at 37 °C. The plates were further processed the same as the standard ELISA with the exception of using streptavidin-HRP for those mAbs that were biotinylated. All plates were read at 450 nm.

### 2.6. Biacore Surface Plasmon Resonance (SPR) Analysis of mAb Binding to V-Antigen

The specific kinetic interactions or affinities between full-length V-antigen and anti-V mAbs was accomplished using an antibody capture approach on a Biacore 3000 SPR instrument (Cytiva, Marlborough, MA, USA). First, a polyclonal rabbit anti-mouse Fc γ was immobilized on the surface of a sensor CM5 chip at a high density (~10,000 RU) using the amine coupling method. Next, each monoclonal antibody was injected at a concentration of 200 nM and a constant flow rate of 20 µL/min until approximately 300 RU/flow cell of each antibody was captured (approximately 7.5 min). In order to remove poorly captured mAbs, the chip surface was rinsed with buffer for 10 min at 20 µL/min. For binding experiments, different amounts of V-antigen (1 nM to 1.5 uM) were passed over the captured anti-V mAb and over a control surface comprised of immobilized anti-mouse Fc antibody (these baseline signals were subtracted from signals of V binding to captured anti-V mAb) at a rate of 20 µL/min for 3 min. The dissociation phase was followed for 10 min, and the surface was regenerated with 10 mM EDTA, pH 8.0, and 2M NaCl between each concentration of V-antigen. The binding phase was used to determine the association constant (ka) between each mAb and V-antigen. The dissociation phase (kd) was measured using the rate of decline in RU on introduction of buffer at the end of injection of V-antigen. Data were reference subtracted and analyzed using BiaEvaluation Software (Cytiva, version 4.1, Marlborough, MA, USA). Kinetic data was fitted to 1:1 Langmuir binding models.

The evaluation of epitope binding of anti-V mAbs to the V-antigen by was done by a competitive assay. A high density polyclonal anti-mouse Fc was first immobilized to flow cells one and two of a research grade sensor CM5 chip (Cytiva, Marlborough, MA, USA). All injections were performed for 4 min at 20 µL/min. A single mAb, 141-1b (biotinylated), was injected over the surface at a concentration of 200 nM. This was followed by a blocking step using a 1:20 diluted normal mouse serum. The blocking step was done to ensure that the remaining anti-mouse Fc sites, not bound to 141-1b, were not available for binding by other mAbs. Following blocking, V-antigen was injected at a concentration of 100 nM, and the remaining mAbs (200 nM) were injected in succession, without regeneration between cycles.

### 2.7. Statistics

Survival curves were compared by Log-Rank test. EC_50_s were estimated under a four-parameter logit model, and the log transformed EC_50_s were compared amongst groups by *t*-test. Absorbance measures were similarly log transformed prior to applying a *t*-test, with results summarized as geometric mean and geometric standard error. Rate constants were compared between groups by forming a *z*-statistic, taken as the difference between groups, divided by the square root of the sum of their squared standard errors. Analyses were conducted using SAS VER 9.4 (SAS Institute, Cary, NC, USA).

## 3. Results

### 3.1. Potency of Anti-Y. Pestis mAb 7.3

We wanted to know how potent anti-*Y. pestis* mAb 7.3 was in a passive protection study in BALB/c mice challenged with wild-type *Y. pestis* CO92. To answer this question mice were inoculated i.p. with varying concentrations of mAb 7.3 (1.0 µg to 500 µg) 24 h before they were challenged s.c. with *Y. pestis* CO92 (21 LD_50_) in a bubonic plague model. A negative control group (anti-*Burkholderia mallei* mAb), and a positive control group (rabbit anti-V antibody Rab 3–89) were also included. The results (Table 1) showed that 28 days after challenge 100% of the mice were fully protected by 500 µg and 50 µg of mAb 7.3 (mean time to death, MTD, >28 days), and most of the mice (6/8) were protected by only 10 µg of mAb 7.3 (MTD, 14.5 days). At 5.0 µg and 1.0 µg of mAb 7.3, there were only 1 (MTD, 15.1 days) and 0 (MTD, 7.3 days) number of survivors, respectively. There was a significant difference in the number of survivors between the groups that received 500 µg and 50 µg of 7.3 mAb when compared with the number of survivors in the groups that received 5.0 µg and 1.0 µg of 7.3 mAb (*p = 0.0061*, and *p < 0.0001*, respectively).

### 3.2. Isolation of Protective Anti-V mAbs

Anti-V mAbs were produced by standard procedures, and hybridoma cell culture supernatants were tested for efficacy in a mouse model of plague. Table 2 shows the results of a select number of anti-V mAb clones (5/19) that were evaluated for their potential to passively protect BALB/c mice from an aerosol exposure to *Y. pestis* C12, the capsule negative strain of the plague organism [24]. Six mice per group were given 5.0 mg of hybridoma culture supernatant of the selected clone intraperitoneal (i.p.) the day before challenge. Control mouse groups included those given a nonprotective anti-V mAb (7H12), and another group of mice given a rabbit anti-V antiserum (Rab 3–89).

The inoculated mice were challenged by aerosol (25 LD_50_), and the number of survivors were followed for 15 days. The number of survivors given the anti-V mAb ranged from 3/6 to 4/6 after 15 days, while all mice (6/6) given the rabbit anti-V Rab 3–89 serum survived the challenge.

**Figure 1:**
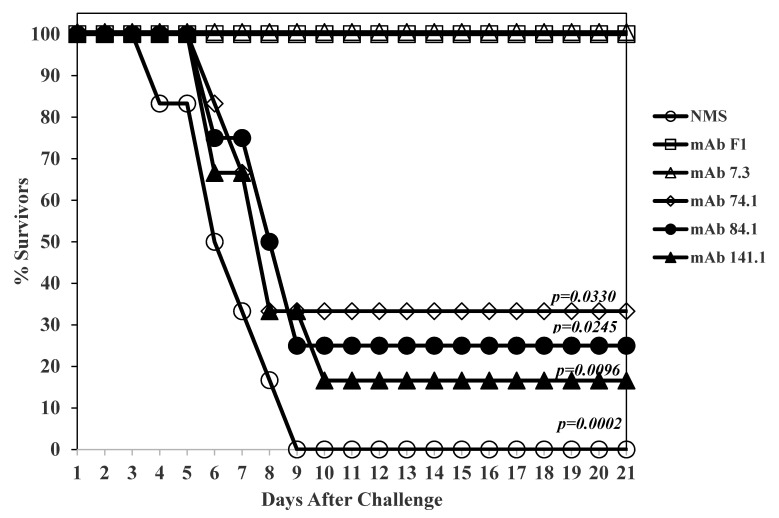
Passive protection conferred by purified anti-V-antigen mAbs in Swiss-Webster mice using a bubonic plague model. There were 4 or 6 mice per group with 6 groups that consisted of the anti-V mAbs (74.1, 500 µg; and 141.1, 500 µg), with 3 control groups comprising normal mouse serum (NMS), anti-V mAb 7.3 (100 µg), and anti-F1 capsule mAb (250 µg). There were only 4 mice in the group that received mAb 84.1. This study was performed 2 times with BALB/c mice (where no protection was observed) and only once with Swiss Webster mice which it shown here. Mice were given the mAb [or Normal mouse serum (NMS)] i.p. 24 h before challenge. Mice were then challenged with 38.5 LD_50_ (1 LD_50_ ~2 CFU) [27] of *Y. pestis* CO92 s.c. and observed for 22 days. The *p* values are shown compared with the number of survivors with mAb 7.3.

After the selected anti-V mAbs were purified, they were re-evaluated for efficacy in a bubonic plague model in BALB/c mice. However, after two attempts, we could not replicate the protection with the culture supernatants we had seen previously in BALB/c mice. The purified anti-V mAbs were then evaluated in Swiss Webster mice. Figure 1 shows the results with anti-V mAbs 74.1, 84.1, and 141.1. The anti-V mAb 7.3 was also evaluated under the same condition as a positive control, as well as an anti-F1 (capsular) mAb [18]. A negative control group received an equivalent amount of normal mouse serum (NMS). Twenty-one days after a *Y. pestis* CO92 s.c. challenge (38.5 LD_50_), there were 2/6 (74.1), 1/4 (84.1), and 1/6 (141.1) survivors in our anti-V mAbs groups, but all mice that received anti-V mAb 7.3 or anti-F1 mAb survived (6/6) (Figure 1). The mean time to death (MTD) for the non-survivors were 6.5 days (NMS), 8.0 days (74.1), 8.5 days (84.1, and 8.0 days (141.1). The MTD for our non-survivors compared to that of the NMS group was not significantly different, but when compared with the mice that received anti-V 7.3 or anti-F1 mAbs the differences were significantly different (see Figure 1). Thus, we isolated anti-V mAbs that gave limited protection to *Y. pestis* CO92 in a bubonic plague model when compared to mAb 7.3.

### 3.3. Comparison of the Binding Sites of the Protective Anti-V mAbs with that of Anti-V 7.3 mAbs on the V-Antigen

The recognition site of the anti-V 7.3 mAb was reported to be a conformational site located between amino acids 135 to 275 on the V-antigen [19]. We asked if this was important for the efficacy of a protective anti-V mAb. Accordingly, we mapped the binding sites of our anti-V mAbs that showed some protection and compared it to that of the 7.3 mAb on the V-antigen. A 53 linear overlapping peptides bank that spanned the length of the V-antigen was first used. An ELISA assay with the individual linear peptide was used as the capture antigen to evaluate binding. The results of the binding by anti-V mAbs are shown in Supplement Figure S1. The binding was examined at least twice in triplicate. We saw binding by the anti-V mAb 10.1 (albeit low) to peptides 1 and 2, by mAb 74.1 to peptides 5 and 6, by mAb 84.1 to peptides 1 and 2, and by mAb 125.1 to peptides 43, 44, and 45. The complete V-antigen was used as a positive control (well 54) to monitor binding. We did not see binding by mAbs 141.1 or 7.3 to the linear peptides, while binding could be detected to the complete V-antigen (Figure S1, panels 4 and 5, respectively). As a control mAb for the V-antigen peptides, the binding of a protective anti-F1 mAb was examined on a 27 peptide bank of the F1 antigen that showed it bound to peptides 1 and 2 that were located at the amino terminal end of the F1 capsular antigen, and the binding of anti-F1 mAb was examined on the 53 peptide bank of the V-antigen as a control (Supplement Figure S2).

### 3.4. Binding of Anti-V mAbs and 7.3 to Regions of a Truncated V-Antigen

**Figure 2:**
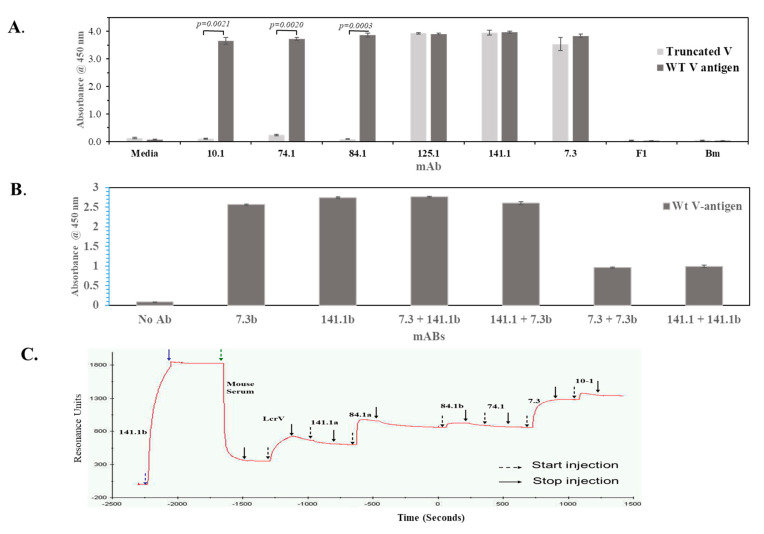
Binding of anti-V mAbs on the wild-type V-antigen or the truncated V-antigen. (**A**). The wild-type or truncated (135–275 amino acids) V-antigen were used at 10 µg/mL, and the mAbs were used at 10 µg/mL to examine binding. Negative control mAbs used were anti-F1 capsule and anti-*B. mallei*, besides without any anti-V mAb (media only). This assay was done twice in triplicate, and a representative assay is shown. Significant differences were only seen between the amount of binding between the wild-type V-antigen and truncated V-antigen with mAbs 10.1, 74.1, and 84.1, but not with mAbs 7.3, 141.1, and 125.1. (**B**). Competitive binding was performed to assess if mAb 7.3 and 141.1 bound to the same site on the V-antigen. The wild-type V-antigen was used at 2 µg/mL, and each mAb was added at 0.1 µg/mL whether it was single or with another mAb. The secondary detection antibody was a streptavidin conjugated HRP to measure the amount of biotinylated mAb bound to the V-antigen. The b added to the mAb designates it as a biotinylated mAb (141.1b or 7.3b). This assay was done twice in triplicate. Comparisons were made between the amount of binding of mAb 7.3b and with that of the other mAb and combinations. All were significantly different (*p < 0.0001*) except with 141.1 + 7.3b. (**C**). Surface plasmon resonance (SPR) analysis was used to examine binding of mAb 141.1 and 7.3 on the V-antigen. The mAb 141.1b (suffix b, for biotinylated) was first captured by a polyclonal anti-mouse Fc γ that was immobilized on a CM5 sensor chip. After blocking the complex with normal mouse serum, the V-antigen (100 nM) was captured by mAb 141.1b immobilized on the chip. Subsequently, other mAbs both biotinylated and unbiotinylated (designated with the suffix, a) (200 nM) were injected in succession without regeneration between mAbs. The SPR binding analysis was performed once. Efficient binding of mAb 74.1 to the V-antigen was not detected in the SPR assay (see Figure S4).

Another approach that was used to evaluate binding of the anti-V mAbs to regions of the V-antigen was to examine binding to a truncated V-antigen that consisted of amino acids 135 to 275, similar to that used by Hill et al. (1997) [18]. The binding of the mAb to the truncated V-antigen and full-length V-antigen were compared to confirm the results of the binding to the V-antigen peptides. This was done twice in triplicate using an ELISA with the two V-antigens as the capture antigen. Figure 2A shows that while we detected binding to the whole V-antigen by all the mAb that were examined, only mAbs 125.1, 141.1, and 7.3 were able to bind to the truncated V-antigen. Our results showed that mAbs 141.1 and 7.3 could bind to the truncated V-antigen but not the linear V-antigen peptides, while mAb 125.1 could bind to both. Thus, the results suggested that mAbs 141.1 and 7.3 appear to bind a conformational site on the V-antigen.

### 3.5. mAbs 141.1 and 7.3 do not Bind to the Same Conformational Site on the V-Antigen

Since mAb 141.1 and 7.3 appear to bind a conformational site located within amino acids 135 to 275 on the V-antigen, it was then asked if they bind to the same conformational site. Two methods were used to answer this question. First, an ELISA based competition assay with mAbs 141.1 and 7.3 was performed. An aliquot of each mAbs was biotinylated to generate a form of the mAb that could be measured when a mixture of unlabeled and biotin labeled mAbs (designated by the suffix b) were used. A streptavidin-HRP was used to measure the presence of the biotinylated form of the mAb. This competition assay was performed twice in triplicate. Figure 2B shows an example where the biotinylated mAb 7.3b and 141.1b could bind to the full-length V-antigen. Also, when unbiotinylated mAb 7.3 and 141.1 were mixed with the biotinylated form of mAb 141.1b and 7.3b, respectively, no inhibition of binding was observed. However, when unlabeled mAb 7.3 or 141.1 was added with the corresponding biotinylated mAb, we saw at least half of the binding of the respective biotinylated form of the mAb. This latter result suggests that the competition assay was working and that the mAbs 141.1 and 7.3 do not appear to bind the same conformational site on the V-antigen.

**Figure 3:**
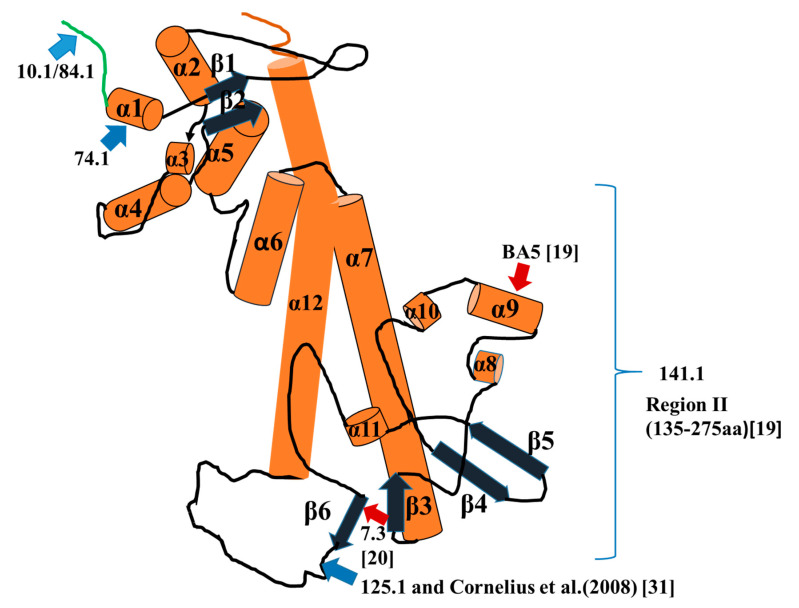
Schematic diagram of binding sites on the V-antigen. The structure of the V- antigen as shown is based on that reported by Derewenda et al. (2004) [30]. The orange tubular structures represent coiled-coil peptides, and the dark blue structures represent β-sheets with arrow heads pointing in the 5′ to 3′-direction. The red arrows are pointing to reported or putative binding sites for mAb 7.3 [21] and BA5 [20]. The light blue arrows are pointing to the binding sites of mAbs (10.1/84.1, 74.1, and 125.1) from this report, except the binding site for 125.1 also represents that reported by Cornelius et al. (2008) [31] for polyclonal antibodies induced by a nonhuman primate and BALB/c mice by the wild-type V-antigen [31]. The amino-terminal end of the protein is colored green, while the C-terminal end is colored red. The conformational binding site of 141.1 is unknown but present on the 135–275 amino acid protein fragment or in designated region II of the V- antigen [19].

In another method to examine binding of mAbs 141.1 and 7.3 to the V-antigen, we used Biacore surface plasmon resonance (SPR) technology. A biotinylated form of 141.1b was first captured by an anti-mouse Fc that was immobilized on a chip in a flow cell. After washing and blocking with normal mouse serum V-antigen was added and captured by the immobilized mAb 141.1b. Then test anti-V mAbs were injected into the flow cell in succession without regeneration between cycles. Although we evaluated the binding of other mAb (as control mAbs), we focused our studies on the binding of mAbs 141.1 and 7.3. The results in Figure 2C showed that V-antigen bound very well to the immobilized mAb 141.1b. We did not detect binding by unbiotinylated mAb 141.1a (no change in resonance units). Monoclonal antibody 84.1a (unbiotinylated) showed good binding but not subsequently by mAb 84.1b. Monoclonal antibody 7.3 bound well to the mAb 141.1b captured V-antigen. There was some minor binding to the captured V-antigen by mAb 10.1, which concurred with our results from the linear V-antigen peptide array that suggested that mAbs 84.1 and 10.1 occupied the same two peptides at the NH-terminal end of V-antigen. We could not detect binding by mAb 74.1 under these conditions, unexpectedly (see Figure S4). Hence, the Biacore SPR capture assay suggested that mAbs 141.1 and 7.3 occupied different conformational sites on the V-antigen. Figure 3 shows a summary of the binding of the mAbs to the V-antigen. Also, included were the putative binding sites of mAb 7.3 [19,21], and another protective anti-V-antigen mAb BA5 [20]. It also not known at this time if mAb 125.1 that binds near the 3′-end of the 135–275 amino acid fragment could interfere with the binding of mAb 7.3 or the other way around since the putative binding sites of the two mAbs appear to be relatively close. It was not clear if mAbs 10.1 and 84.1 are identical, but they are different in some binding properties (see below). Only the location of the conformational binding site of mAb 141.1 was not clear at this time although it was within the 135–275 amino acid fragment of the V-antigen.

### 3.6. mAb 7.3 Does not Have a Higher Avidity or Affinity to the V-Antigen than Less Protective mAbs

**Figure 4:**
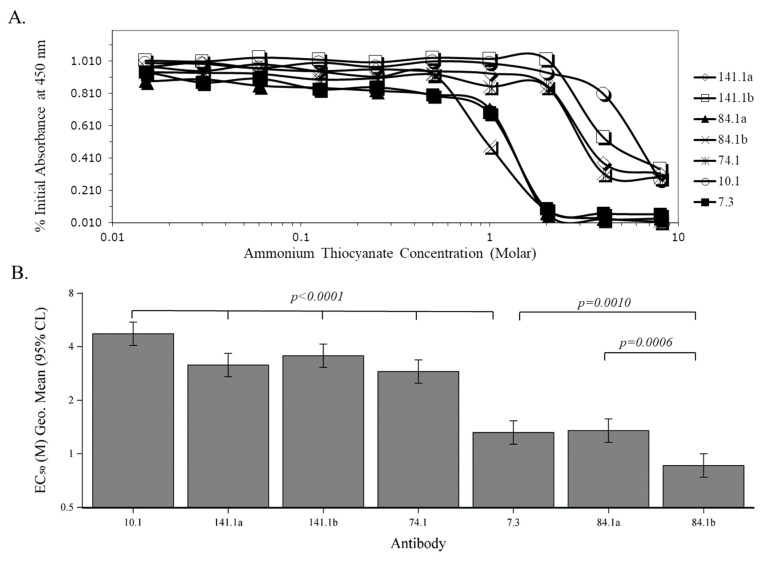
Evaluation of the avidity of anti-V mAbs to the V-antigen. (**A**). The avidity of anti-V mAbs to the V-antigen was measured in increasing concentrations of ammonium thiocyanate concentrations. After the binding of the anti-V mAbs to the V-antigen the mAb-V-antigen complex was incubated in increasing concentrations of ammonium thiocyanate (0.015 M to 8 M) for 30 min at 37 °C. Anti-V mAbs 7.3, 10.1, 74.1, 84.1 and 141.1 were examined under these conditions. In some cases, unbiotinylated (designated with an “a” suffix) and biotinylated (designated with a “b” suffix) mAbs were examined. This assay was performed 3 times in triplicate, and one of the examples is shown here. (**B**). The EC_50_ of the avidity assay comparing the anti-V-antigen mAbs is shown. The differences between the EC_50_ of mAb 7.3 to the other mAbs was compared, and the difference was *p < 0.0001* with mAb 10.1, 141.1a, 141.1b, and 74.1. When the EC_50_ of 7.3 was compared with that of 84.1a and 84.1b, there was only a difference with 84.1b (*p = 0.0010*) but not with 84.1a.

Since we had shown that our anti-V mAbs do not bind to the V-antigen at the same site as mAb 7.3, we asked if the avidity or affinity in binding of mAb 7.3 to the V-antigen could be responsible for the better protection provided by the mAb to a lethal *Y. pestis* challenge. We used two different methods to examine the binding interaction of the anti-V mAbs to the V-antigen. In the first method, we measured the avidity between the mAbs and V-antigen by subjecting the mAb -V-antigen complex to increasing ammonium thiocyanate concentrations (0.008–8.0 M) and measured the amount of binding left in an ELISA type format. The same anti-V mAbs (unbiotinylated and biotinylated forms) used previously were examined and this assay was repeated three times in triplicate. Figure 4A shows a typical result of one of the binding assays with the mAb-V-antigen complex in the presence of increasing concentrations of ammonium thiocyanate. In Figure 4B, we reported the median effective dose (EC_50_) of the individual mAbs derived from the titration studies. There appears to be two general mAb groups: 1. higher avidity, 10.1, 141.1a, 141.1b, and 74.1; and 2. lower avidity, 7.3, 84.1a, and 84.1b. All the higher binding mAbs were significantly higher than mAb 7.3 (*p* < 0.0001), while in the lower binding mAbs, only 84.1b was different than mAb 7.3 (*p* = 0.0010). Biotinylation also did not change the strength in binding between mAb 141.1a and 141.1b, although we saw differences between mAb 84.1a and 84.1b (*p* = 0.0006). The results in the latter case suggest that biotinylation of specific amino acids at or near the binding site of 84.1 increased the affinity for the V-antigen, but not in the case for 141.1 in this assay.

The second method that was used to evaluate the binding of the anti-V mAbs to the V-antigen was by kinetic analysis (affinity) using Biacore SPR technology. After each mAb was captured on a flow cell a series of V-antigen concentrations ranging from 1 nM to 1.5 uM was passed over the flow cell with a regeneration step after each concentration of V-antigen (see Figure S3). The same mAbs used in the previous ELISA avidity study were used, but the mAb 74.1 was not active in the Biacore SPR assay. As in the previous study, mAbs with the suffix “a” were unbiotinylated, while mAbs with the suffix “b” were biotinylated. As with the avidity study, there were two general affinity groups of mAbs; a low affinity group (10.1, 84.1a, and 84.1b), and a high affinity group (7.3, 141.1a, and 141.1b) (Table 3). The K_D_s of the low affinity mAbs ranged from 10.7 to 25.4 nM, while those in the high affinity group ranged from 0.77 to 4.03 nM. There was also a difference (*p* < 0.0001) in affinity between the unbiotinylated and biotinylated mAb (84.1 and 141.1). In the case with mAb 84.1, biotinylation decreased the affinity for V-antigen, but for mAb 141.1, biotinylation enhanced the affinity for V-antigen. Thus, although the most protective mAb 7.3 fell within the high affinity group (4.05 nM), it did not exhibit the highest affinity for the V-antigen in the group.

## 4. Discussion

The present study demonstrated that the overall interaction of mAb 7.3 with the V- antigen did not appear to be more exceptional than other partially protective mAbs, nevertheless, mAb 7.3 was the most protective mAb against infection by *Y. pestis* CO92 in a murine plague model. This was found after our comparison of partially protective mAbs with that of mAb 7.3 with the avidity to V-antigen or the affinity for the antigen that might predict the efficacy of mAb 7.3 against plague infection. Hence, it was concluded that it could be the binding site of mAb 7.3 that was most critical for its efficacy. It has been reported that mAb 7.3 binds to a conformational site of V-antigen between amino acids 135–275 [19,32]. This was confirmed in our own studies that mAb 7.3 did not bind to a linear V-antigen peptide library, but it could bind to a full-length V-antigen or a V-antigen fragment consisting of amino acids 135–275. Our results also demonstrated that mAb 141.1, which provided partial protection, recognized a conformational site on the V-antigen fragment that was different from the site used by mAb 7.3.

**Figure 5:**
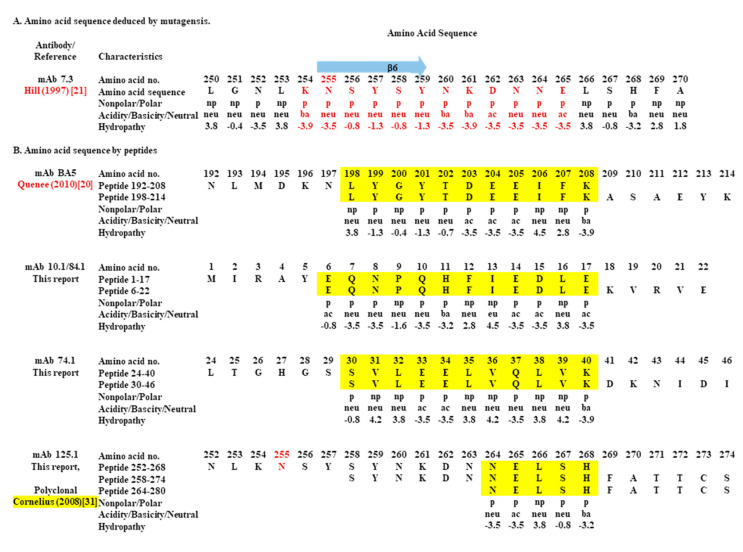
Summary of amino acids in putative binding sites in the V-antigen derived from mutagenesis or mAbs binding to linear peptides. (**A**). Region of binding of mAb 7.3 derived from mutational analysis identified N255 as a critical amino acid [21]. The region from amino acid 250 to 270 is shown with N255 in red and the beta-sheet β6 shown above. Also listed in red are amino acids 254–265 that includes N255, and polarity (nonpolar, np/polar, p), acidity (ac)/basicity (ba)/neutrality (neu), and hydropathy index. Note 12 contiguous negative hydropathic amino acids. (**B**). Region of common overlapping V-antigen peptides identified by binding by mAbs (in yellow). Listed are mAbs BA5 [20] (11 amino acids), 10.1/84.1 (12 amino acids), 74.1 (11 amino acids), and 125.1 (5 amino acids). Also listed are the amino acid number, amino acid, polarity (nonpolar, np/polar, p), acidity(ac)/basicity (ba)/neutrality (neu), and hydropathy idex. The amino acids listed for mAb 125.1 were also identified by another study with polyclonal antibodies from nonhuman primates and BALB/c mice raised against V-antigen [31]. N255 within this region was highlighted in red. The peptide 264–280 amino acids that was recognized by 125.1 was shortened (275–280 amino acids) for presentational purposes in the figure.

An essential amino acid required for protection by mAb 7.3 has been identified at N255, which is located just within the beta-sheet β6 located at the lower end of region II of the V-antigen [21,31]. A mutation at N255 reduced the binding of mAb 7.3 to the mutated V-antigen, and decreased the protection conferred by the wild-type antigen [21]. We examined the primary sequence in the area of the N255 and beta-sheet β6 to see if there were any indications as to why the binding of mAb 7.3 to this region was a requisite for the efficacy of mAb 7.3 (Figure 5A). Examination of amino acids 254 to 265 revealed 12 contiguous polar amino acids that could be part of the conformational binding site for mAb 7.3 and/or the interaction with the proximal anti-parallel beta-sheet β3. The primary sequence of the beta-sheet β3 consists of four amino acids (T183-I-N-186I) which interact with beta-sheet β6 according to the model of the V-antigen structure.

There was also a partial invert repeat (K254-N-S-Y-S-Y-N-K261) that overlapped the beta-sheet β6 region. It has been shown that removal of the region of the V-antigen containing either beta-sheet β3 or β6, eliminates binding of mAb 7.3 [19], suggesting that these structural elements may be involved in the formation and stabilization of the tertiary structure of V-antigen in region II [19]. The V-antigen fragment of amino acid residues 135–275 contains the beta-sheets β3 and β6, and thus the truncated molecule is likely to retain the tertiary structure of the V-antigen molecule in region II. This was suggested by our results that mAb 7.3 could still bind the truncated V-antigen. In addition, the amino acids that are recognized by mAb 7.3 within this region may not be contiguous [33]. The exact mechanism on how mAb 7.3 inhibits the activity of the V-antigen is not clear. V-antigen is a multifunctional protein that is part of the injectisome of the type III secretion system (with *yersinia* Yops B and D) that allows the pathogen to introduce effector molecules into the host cell that suppress the host immune response to the pathogen [34]. The V-antigen can also interact with YopG through a coiled-coil interaction (136–180 amino acids of the V- antigen) [35,36,37]. Antibodies to the antigen can protect macrophages from *Y.* pestis-induced cell death and enhance phagocytosis [38].

In addition to evaluating the binding region of mAb 7.3 on the V-antigen, we have summarized the binding sites identified by partially protective mAbs (10.1/84.1, 74.1, and 125.1) with the linear peptide library of the V-antigen (Figure 5B). Also listed were those sequences identified with the same linear peptide library used in the present study by Quenee et al. (2010) [20] and by Cornelius et al. (2008) [30], who showed that a protective mAb BA5 or a polyclonal antibody induced by recombinant LcrV recognized different regions of the V-antigen. The sequences of the peptides listed (Figure 5B) are those that were recognized by the mAb BA5 and polyclonal antibody, and the common overlapping sequences between the linear peptides is highlighted (yellow). The common overlapping sequences range from 5 to 12 amino acid residues, and there appears to be no common sequences between these linear peptides. The two linear peptides bound by mAb BA5 included 11 common amino acids, however, within these amino acids there are seven contiguous polar amino acids that all exhibit a negative hydropathic index [20]. The V- antigen peptides recognized by mAbs 10.1 and 84.1 are located at the amino terminal end of the V-antigen (peptides 1 and 2), and there are eight out of 12 polar amino acids with nine displaying a negative hydropathic index within the 12 overlapping peptides. There was no similar arrangement of polar amino acids with the peptides recognized by mAb 74.1, or the three linear V-antigen peptides recognized by mAb 125.1, which had the smallest shared sequences (five amino acids) of the mAbs that bound linear peptides. Intriguingly, these identical linear peptides were also recognized by antibodies from BALB/c mice and Cynomolgus macaques vaccinated with the wild-type V-antigen [30]. There was also a contiguous string of 8 polar amino acids (T271-T-S-S-D-K-S-R278) that was located adjacent to the coiled-coil α12 structure of the V-antigen, although we did not see binding of any mAb to the linear peptides in this region [31].

There are some limitations to the comparison of mAb 7.3 to the other antibodies described here that bound the V-antigen. We were not able to isolate a fully protective mAb from *Y. pestis* infection although we started with at least 100 possible clones. Many of these were not further evaluated because they did not produce sufficient anti-V activity, and the purified antibodies were unexpectedly more protective with Swiss Webster than with BALB/c mice. Although mAb 74.1 functioned in other binding studies, this monoclonal antibody did not specifically interact with V-antigen in SPR assays, perhaps because immobilization blocked the binding site. Furthermore, we evaluated the protective ability of the mAbs and 7.3 in a bubonic plague model and not a pneumonic plague model against *Y. pestis* CO92 or *Y. pestis* C12 in BALB/c mice. Passive protection by mAb 7.3 in a pneumonic plague model has been reported previously in BALB/c mice, however, it was against a challenge by *Y*. *pestis* GB [22].

In summary, from our studies of the avidity and affinity of fully and partially protective anti-V antigen mAbs, the results did not reveal why mAb 7.3, which was fully protective against a lethal challenge of Y. pestis CO92, was more protective than the mAbs that gave limited protection. Thus, we concluded that the putative binding site of mAb 7.3 was the most critical property of the protective mAb. The biochemical characteristics of the amino acids within the putative binding site may play a role in the protein secondary or tertiary structure of the V-antigen, and therefore, in the folding and stabilization of the protein, and/or be involved with the activity of the V-antigen. Further genetic and biochemical studies, however, will be needed to determine how important these regions of the V-antigen are for the function of the virulence factor. The present study has further described two critical sites on the V-antigen that can be used for future investigations as potential therapeutic targets against plague.

## Figures and Tables

**Table 1:** Protection of BALB/c mice by *Y. pestis* anti-V monoclonal antibody (mAb) 7.3 from *Y. pestis* CO92 in a bubonic plague model.

mAb	Amount Given (i.p.) ^b^	Challenge Dose No. LD_50_^c^ (s.c.) ^d^	No. Survivors (after 28 Days)	Mean Time to Death (MTD (Days) ^e^
7.3	500 µg	21	8/8	>28
7.3	50 µg	21	8/8	>28
7.3	10 µg	21	6/8	14.5
7.3	5.0 µg	21	1/8 (*p = 0.0061*)	15.1
7.3	1.0 µg	21	0/8 (*p < 0.0001*)	7.3
Bm (Neg Con) ^a^	1.0 mg	21	0/8 (*p < 0.0001*)	7.3
Rab 3–89 (Pos Con)	0.5 mg	21	7/8	12.0

^a^ Bm is an anti-*B. mallei mAb*. This titration study was performed once. ^b^ mAbs were given intraperitoneal (i.p.) the day before challenge. ^c^ 1 LD_50_ is ~2 CFU. ^d^ The challenge dose was subcutaneous (s.c.). ^e^ Mean time to death of mice that died and significant differences compared with MTD 50–500 µg mAb 7.3 groups.

**Table 2:** Protection of BALB/c mice by *Y. pestis* anti-V mAb from an aerosol exposure to *Y. pestis* C12.

	Isotype	Amount Given (mg) ^c^	Challenge Dose (LD_50_) ^d^	No. Survivors (after 15 Days)	MTD (Days) ^e^
10.1 ^a^	IgG1	5.0	25	3/6	5.7
74.1 ^a^	IgG1	5.0	25	4/6	6.0
84.1 ^a^	IgG2a	5.0	25	3/6	6.3
125.1 ^a^	IgG1	5.0	25	3/6	7.3
141.1 ^a^	IgG1	5.0	25	3/6	5.3
7H12 (Neg Con) ^b^	IgG1	3.0	25	1/6	6.0
Rab 3–89 (Pos Con) ^b^	NA	5.0	25	6/6	>15

^a^ Culture supernatant. ^b^ 7H12 is a nonprotective anti-V-antigen mAb, and Rab 3–89 is a rabbit anti-V- antigen serum. ^c^ Antibody was given intraperitoneal the day before challenge. ^d^ 1LD_50_ is approximately 6.8 × 10^4^ CFU. ^e^ Mean time to death of mice that died.

**Table 3:** Affinity of anti-V mAbs to the *Y. pestis* V-antigen determined by Biacore SPR.

mAb ^a^	K_D_ (nM) ± SE	k_a_ × 10^4^ (1/Ms) ± SE	k_d_ × 10^−4^ (1/s) ± SE
10.1	24.3 ± 0.9 ^b^	5.67 ± 0.42	13.8 ± 1.05
84.1a	10.7 ± 0.5 ^b,c^	9.11 ± 0.35	9.66 ± 0.2
84.1b	25.4 ± 1.5 ^b,c^	7.73 ± 0.33	19.3 ± 0.43
7.3	4.03 ± 0.27	16.9 ± 0.47	6.98 ± 0.56
141.1a	2.46 ± 0.06 ^b,c^	22.8 ± 0.49	5.64 ± 0.10
141.1b	0.77 ± 0.06 ^b,c^	40.1 ± 0.51	3.07 ± 0.19

^a^ mAb 74.1 was not functional in the kinetic analysis of binding to the V-antigen (Figure S4). ^b^ Difference in K_D_ relative to 7.3 is *p* < 0.0001. ^c^ There was a significant difference between the K_D_ of unbiotinylated and biotinylated mAbs (*p* < 0.0001).

## Supplementary Materials

The following are available online at https://www.mdpi.com/2073-4468/9/3/37/s1, Figure S1: Binding of anti-V-antigen to linear peptides by anti-V mAbs; Figure S2: Binding of anti-F1 capsule mAb on F1 linear peptides does not show secondary positive bands like with V-antigen peptides; Figure S3: The kinetic binding of V-antigen by anti-V mAbs was analyzed by using an antibody capture method with a Biacore 3000 SPR instrument.; Figure S4: The deficiency of binding of V-antigen by mAb 74.1 was analyzed with a Biacore 3000 SPR instrument.
