# Supplementary material for: Binding Sites of Anti-Lcr V Monoclonal Antibodies Are More Critical than the Avidities and Affinities for Passive Protection against Yersinia pestis Infection in a Bubonic Plague Model
Source: Antibodies (Basel). 2020 Aug 3;9(3):37. doi: 10.3390/antib9030037 (PMC7551159; doi:10.3390/antib9030037)
Supplement: Supplementary file 1 [file antibodies-09-00037-s001.zip › antibodies-818462-supplementary.ppt]

## Slide 1
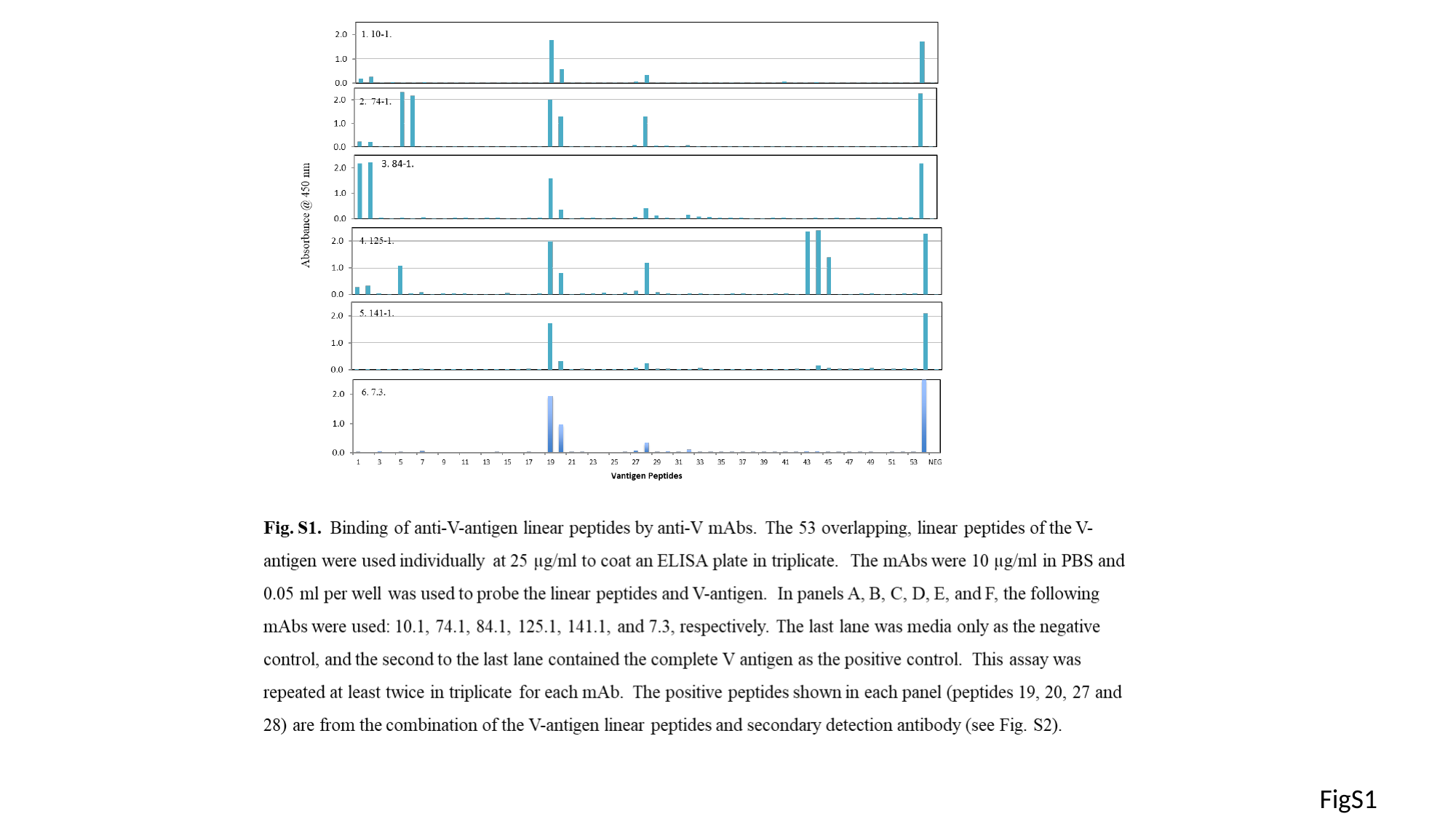

FigS1

## Slide 2
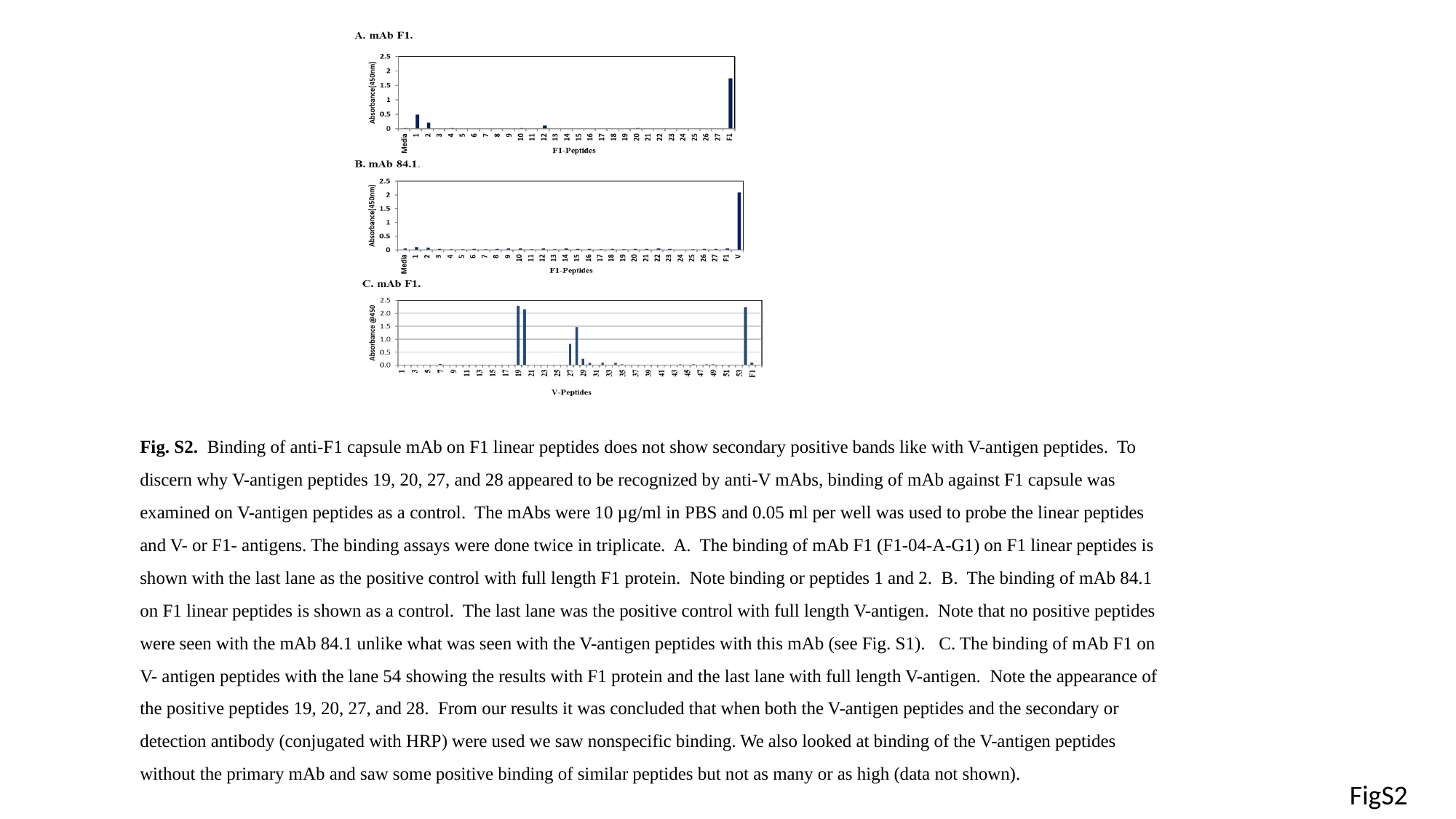

Fig. S2. Binding of anti-F1 capsule mAb on F1 linear peptides does not show secondary positive bands like with V-antigen peptides. To discern why V-antigen peptides 19, 20, 27, and 28 appeared to be recognized by anti-V mAbs, binding of mAb against F1 capsule was examined on V-antigen peptides as a control. The mAbs were 10 µg/ml in PBS and 0.05 ml per well was used to probe the linear peptides and V- or F1- antigens. The binding assays were done twice in triplicate. A. The binding of mAb F1 (F1-04-A-G1) on F1 linear peptides is shown with the last lane as the positive control with full length F1 protein. Note binding or peptides 1 and 2. B. The binding of mAb 84.1 on F1 linear peptides is shown as a control. The last lane was the positive control with full length V-antigen. Note that no positive peptides were seen with the mAb 84.1 unlike what was seen with the V-antigen peptides with this mAb (see Fig. S1). C. The binding of mAb F1 on V- antigen peptides with the lane 54 showing the results with F1 protein and the last lane with full length V-antigen. Note the appearance of the positive peptides 19, 20, 27, and 28. From our results it was concluded that when both the V-antigen peptides and the secondary or detection antibody (conjugated with HRP) were used we saw nonspecific binding. We also looked at binding of the V-antigen peptides without the primary mAb and saw some positive binding of similar peptides but not as many or as high (data not shown).
FigS2

## Slide 3
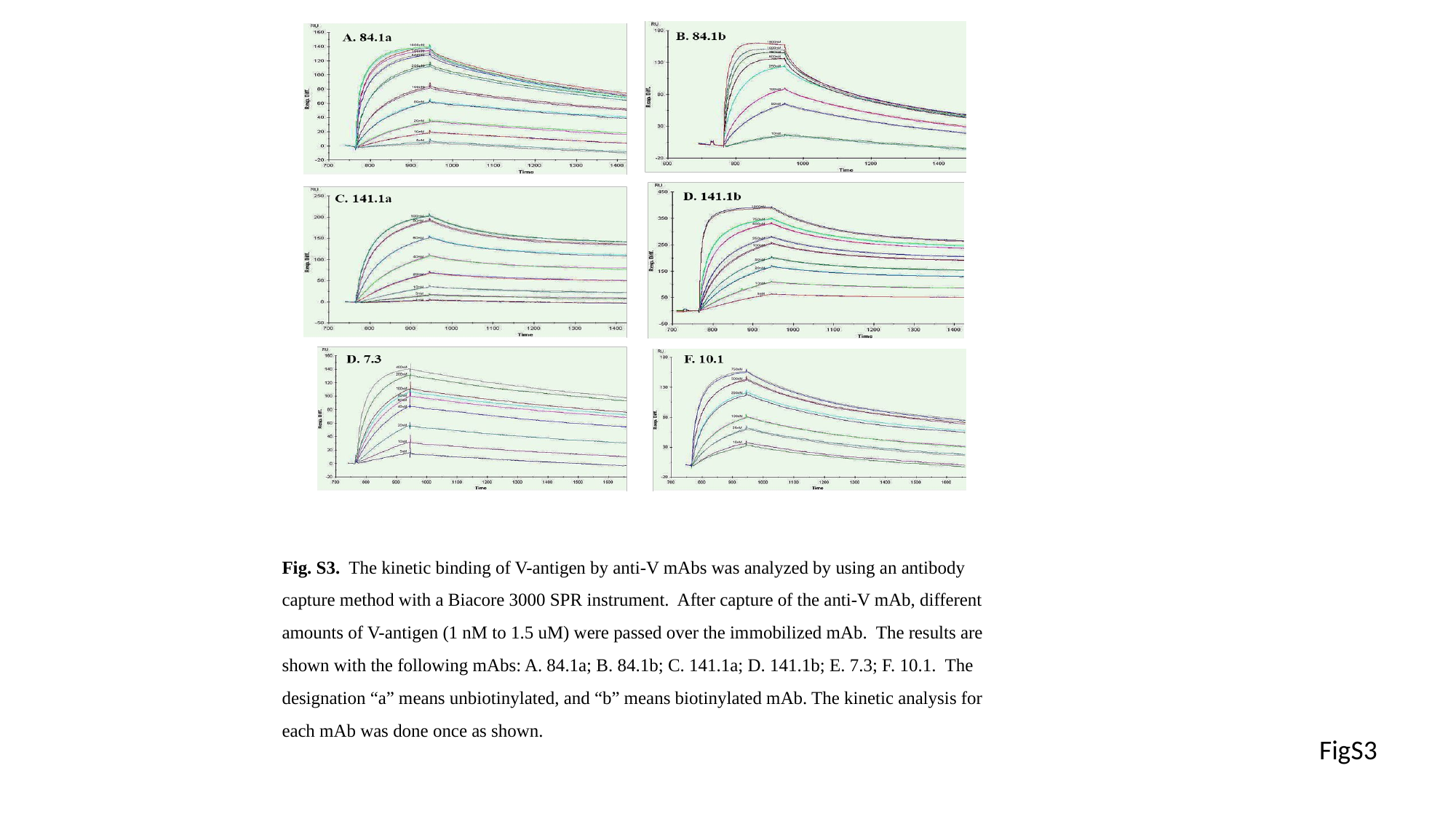

Fig. S3. The kinetic binding of V-antigen by anti-V mAbs was analyzed by using an antibody capture method with a Biacore 3000 SPR instrument. After capture of the anti-V mAb, different amounts of V-antigen (1 nM to 1.5 uM) were passed over the immobilized mAb. The results are shown with the following mAbs: A. 84.1a; B. 84.1b; C. 141.1a; D. 141.1b; E. 7.3; F. 10.1. The designation “a” means unbiotinylated, and “b” means biotinylated mAb. The kinetic analysis for each mAb was done once as shown.
FigS3

## Slide 4
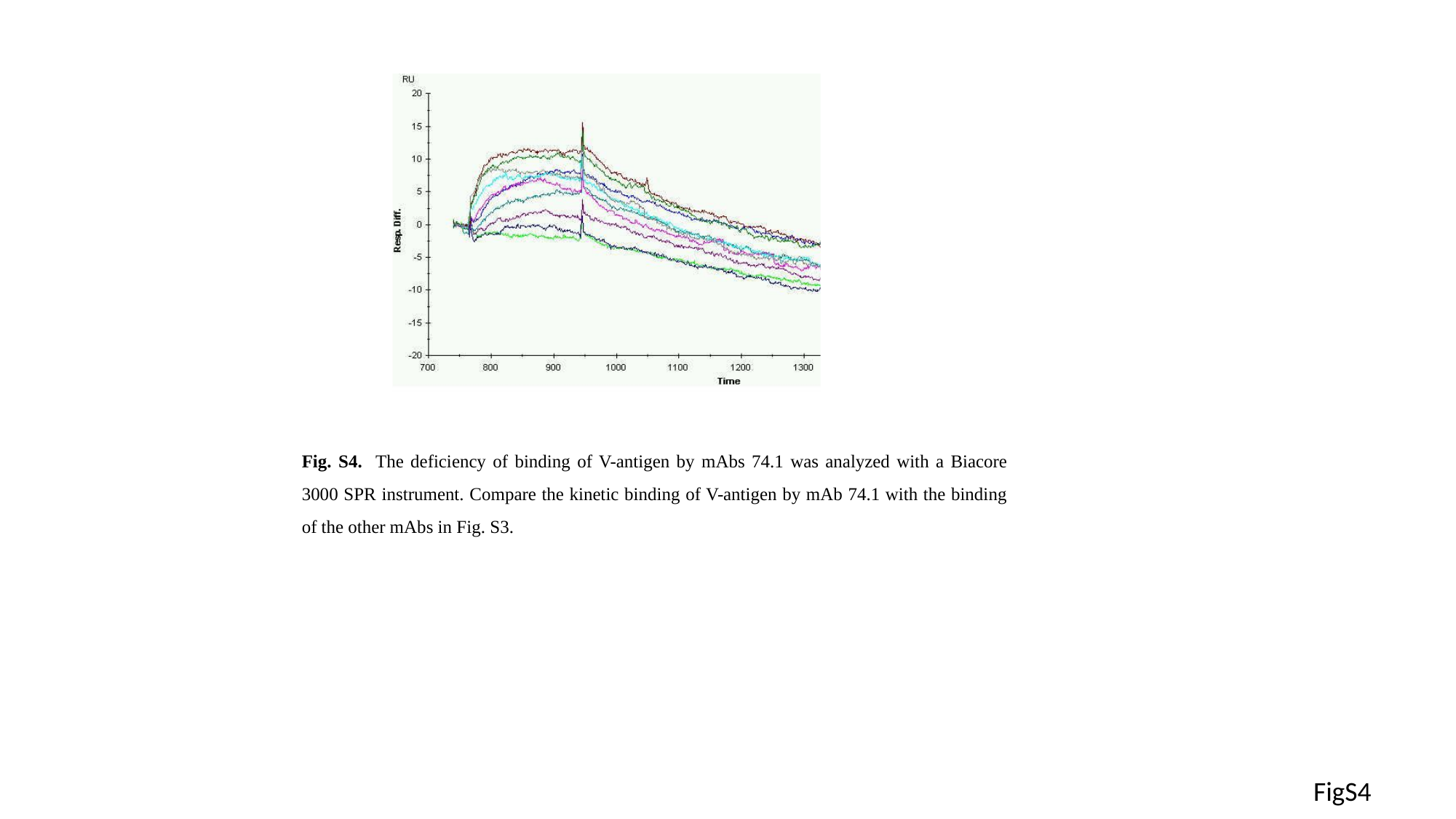

Fig. S4. The deficiency of binding of V-antigen by mAbs 74.1 was analyzed with a Biacore 3000 SPR instrument. Compare the kinetic binding of V-antigen by mAb 74.1 with the binding of the other mAbs in Fig. S3.
FigS4
